# Supplementary material for: Targeting Esophageal Squamous Cell Carcinoma by Combining Copper Ionophore Disulfiram and JMJD3/UTX Inhibitor GSK J4
Source: Cancers (Basel). 2023 Nov 9;15(22):5347. doi: 10.3390/cancers15225347 (PMC10670038; doi:10.3390/cancers15225347)

A. Panel A represents western blot analysis shown in Fig 3E ~ G.

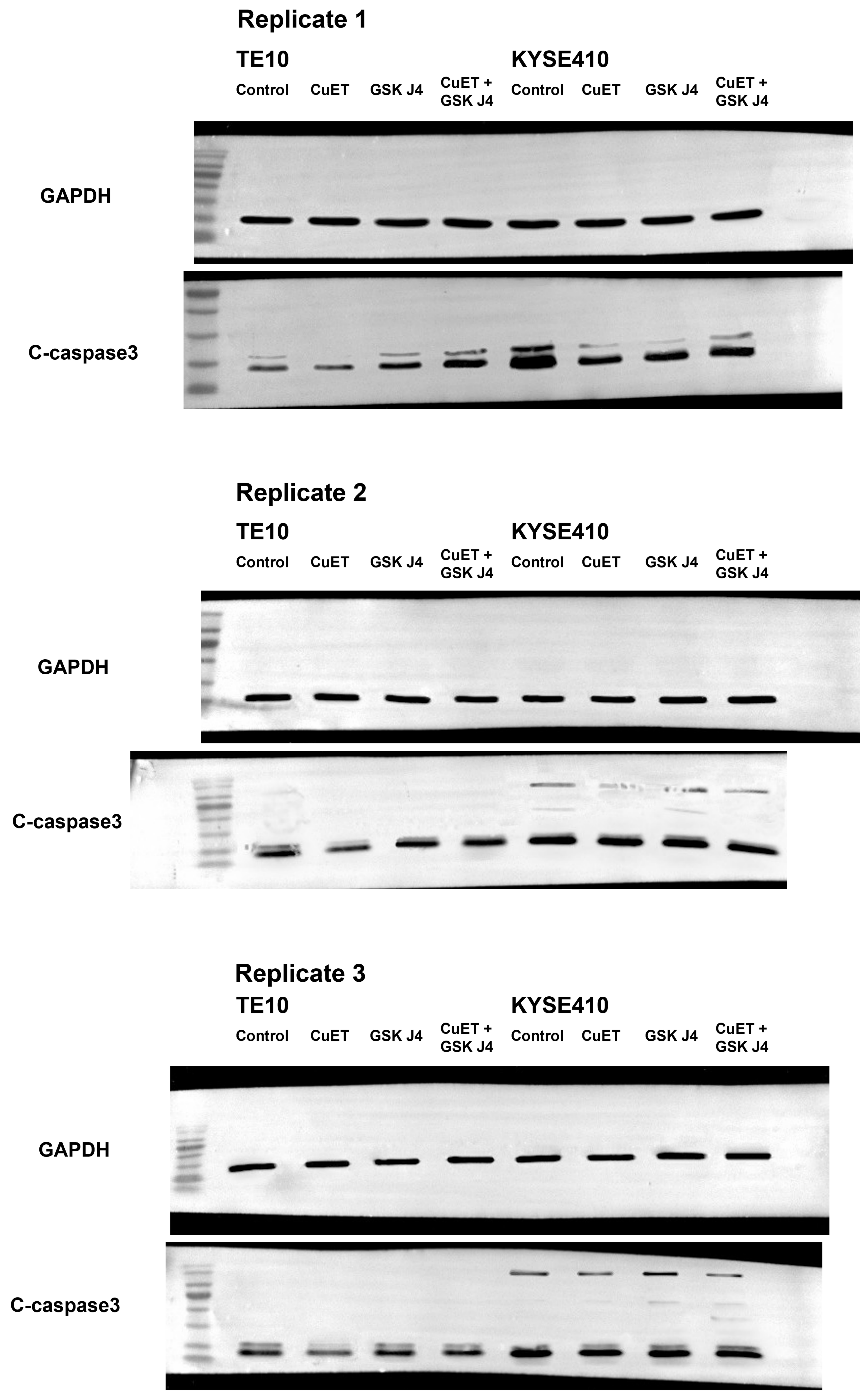

B. Panel B represents western blot analysis shown in Fig 4J ~ M. (The data in the red box was not shown in the article)

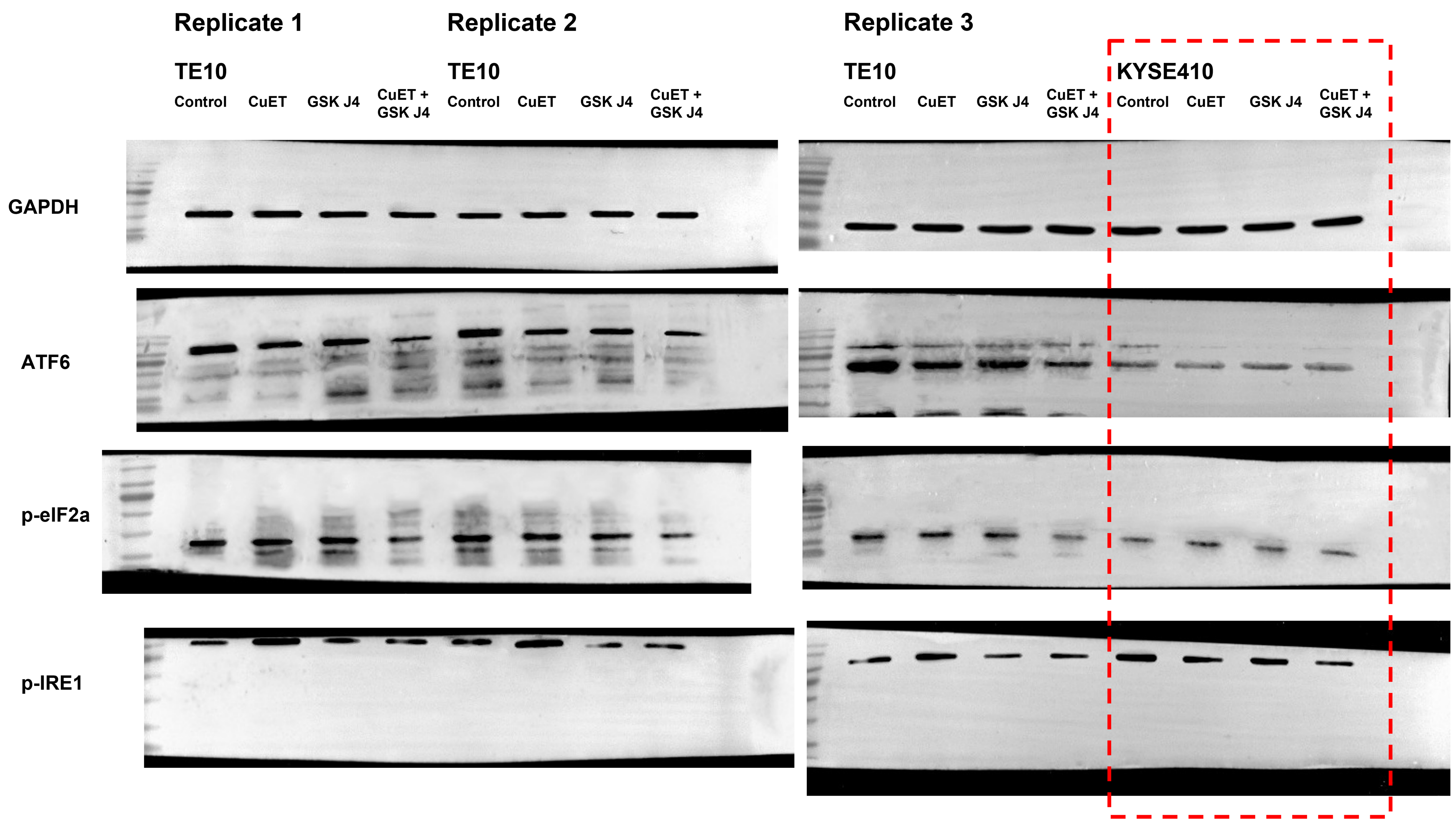

Supplement: Supplementary file 1 [file cancers-15-05347-s001.zip › Fugure S3.pdf]
