# Supplementary figures and images for: Targeting Esophageal Squamous Cell Carcinoma by Combining Copper Ionophore Disulfiram and JMJD3/UTX Inhibitor GSK J4
Source: Cancers (Basel). 2023 Nov 9;15(22):5347. doi: 10.3390/cancers15225347 (PMC10670038; doi:10.3390/cancers15225347)

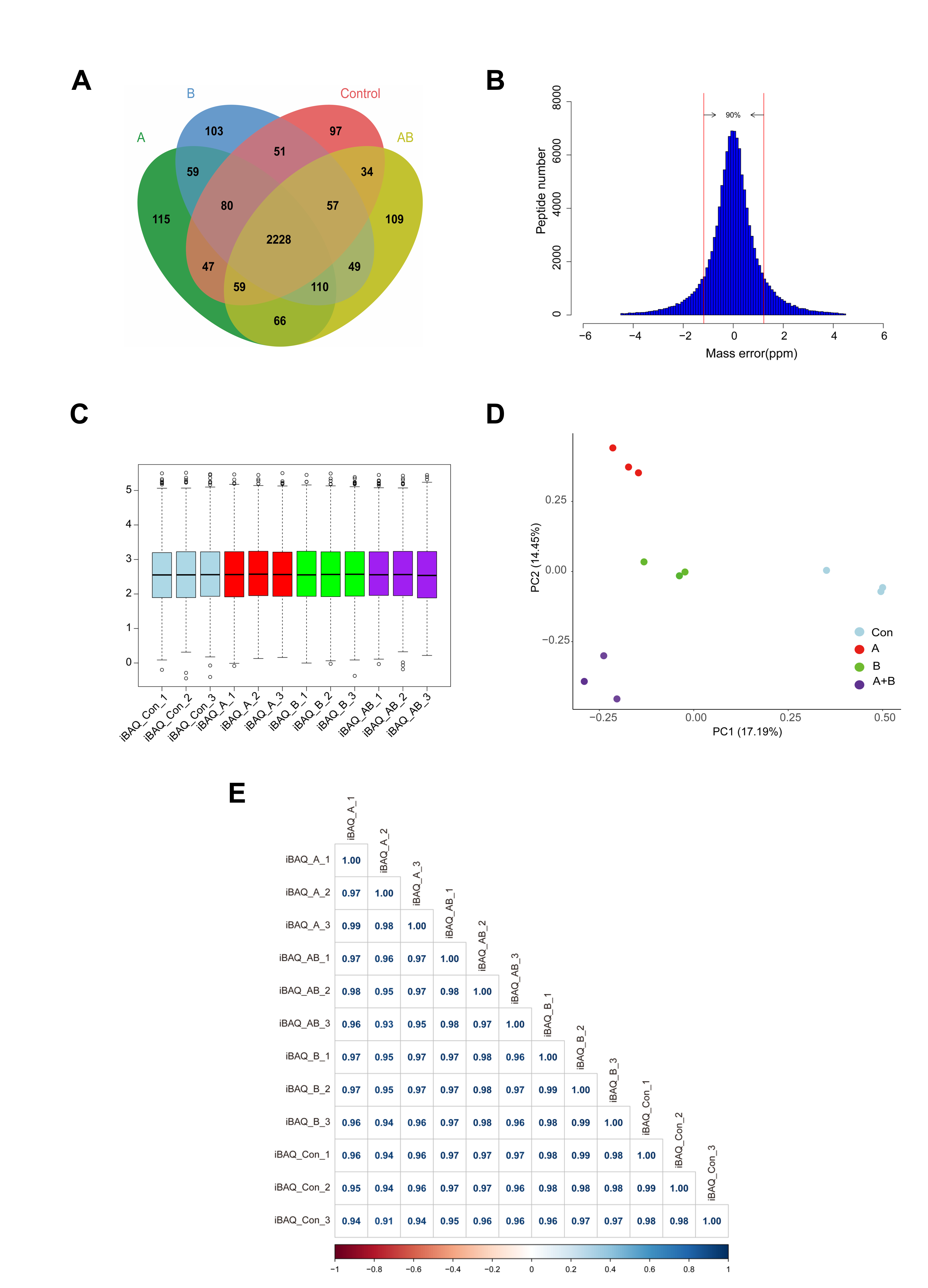

Supplement: Supplementary file 1 [file cancers-15-05347-s001.zip › Figure S1.tif]

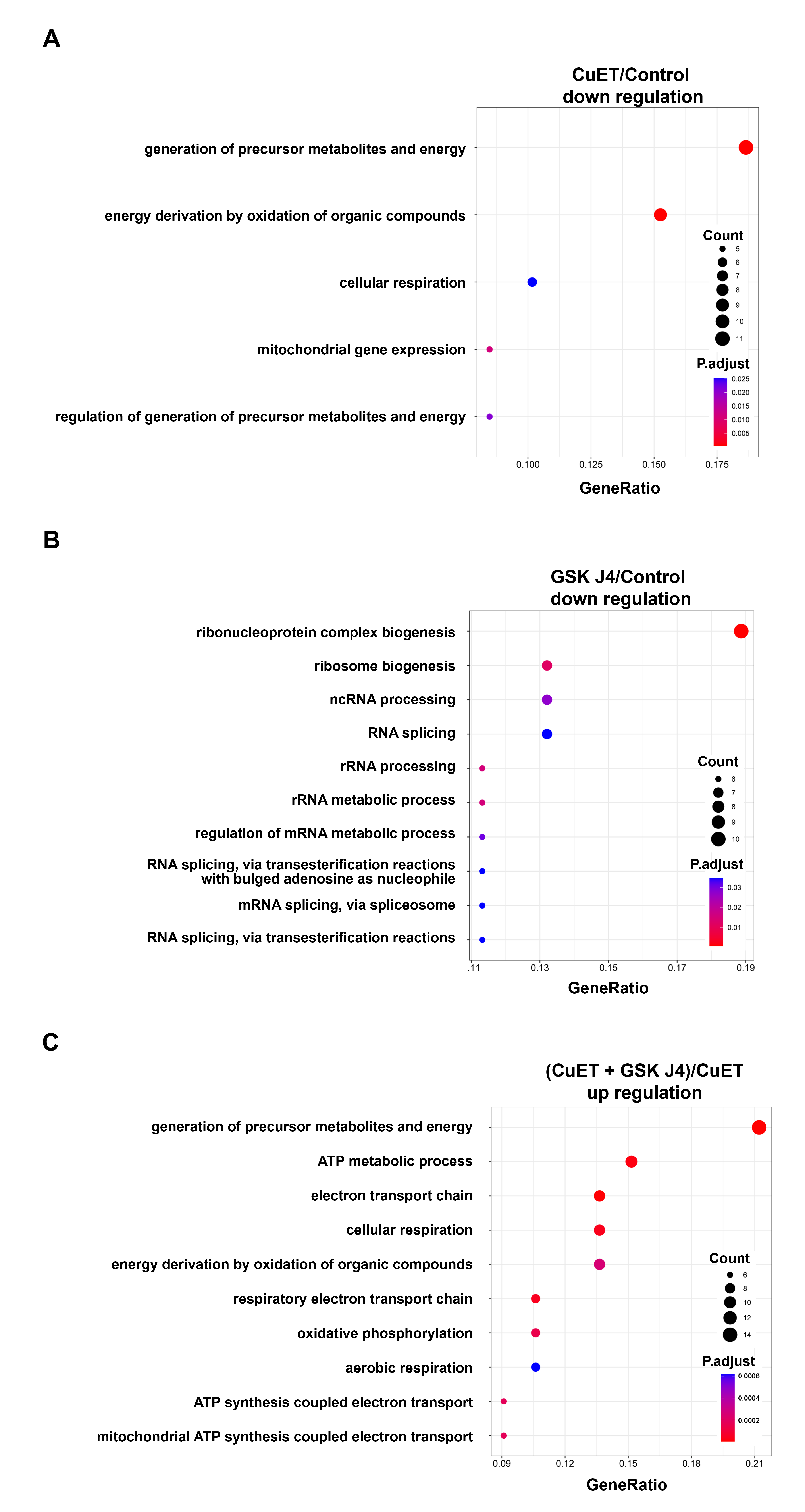

Supplement: Supplementary file 1 [file cancers-15-05347-s001.zip › Figure S2.tif]
